# Supplementary material for: Coupling aqueous zinc batteries and perovskite solar cells for simultaneous energy harvest, conversion and storage
Source: Nat Commun. 2022 Jan 10;13:64. doi: 10.1038/s41467-021-27791-7 (PMC8748727; doi:10.1038/s41467-021-27791-7)
Supplement: Supplementary file 1 — Supplementary Information [file 41467_2021_27791_MOESM1_ESM.pdf]

Supplementary Information

Coupling aqueous zinc batteries and perovskite solar cells for simultaneous energy harvest, conversion and storage

Peng Chen [1] *Tian-Tian Li* [1], *Yuan-Bo Yang* [1], *Guo-Ran Li* [1], *Xue-Ping Gao** [1,2]

[1] Institute of New Energy Material Chemistry, School of Materials Science and Engineering, Nankai University, Tianjin 300350, China.
[2] Renewable Energy Conversion and Storage Center, Nankai University, Tianjin 300350, China.

E-mail: xpgao@nankai.edu.cn

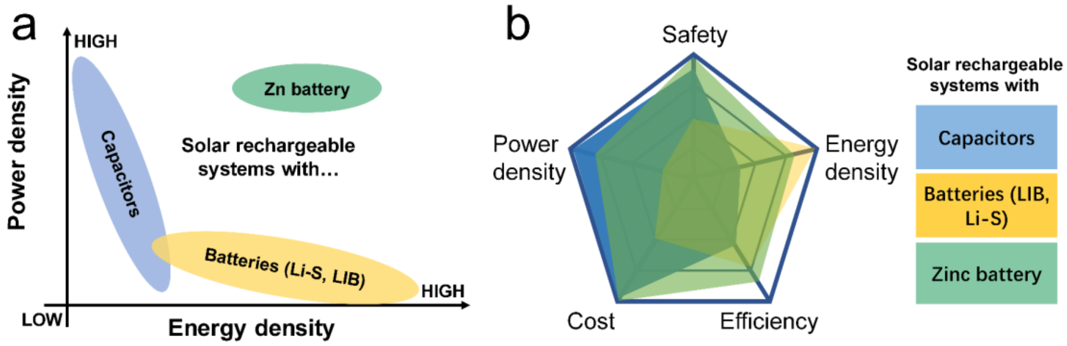

Supplementary Figure 1. (a) Brief comparison of energy density and power density between solar rechargeable systems. (b) Comparison of different solar rechargeable systems of 4H1L principle.

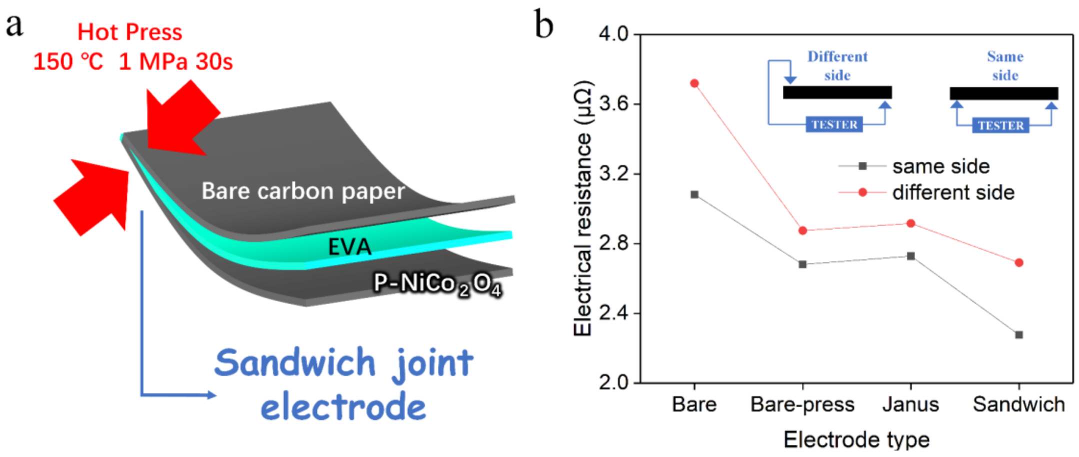

Supplementary Figure 2. (a) Preparation process of Sandwich joint electrode. (b) Electrical resistance tests of different electrodes.

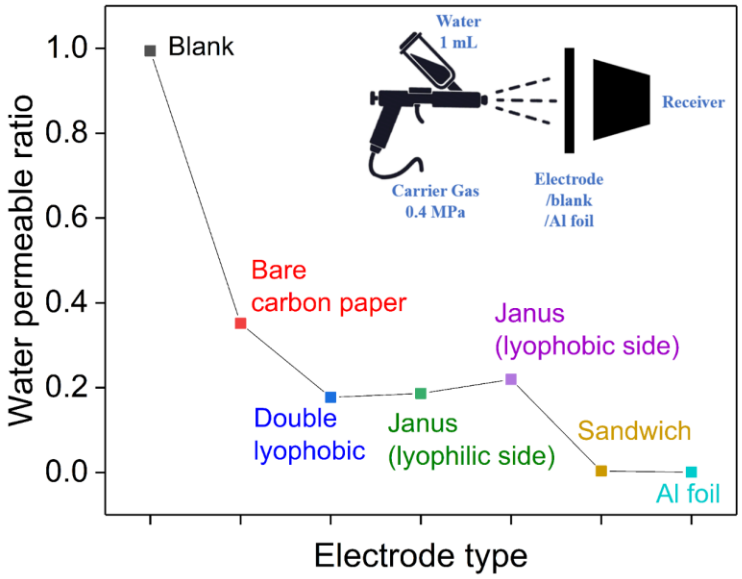

Supplementary Figure 3. Water permeable test of different electrodes.

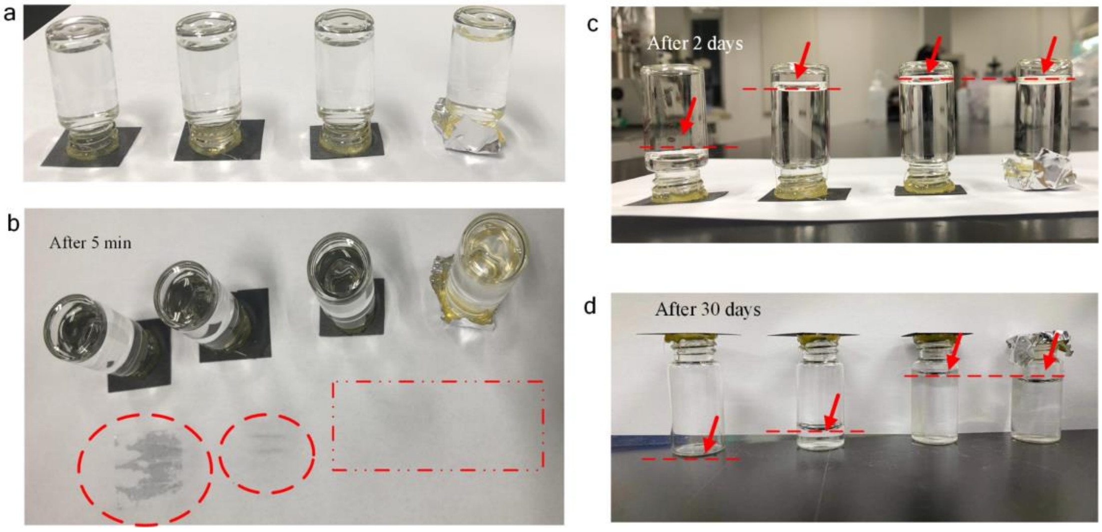

Supplementary Figure 4. Stationary water infiltration tests of four samples sealed by bare carbon paper, Janus carbon paper, Sandwich carbon paper and aluminum foil respectively (from left to right).

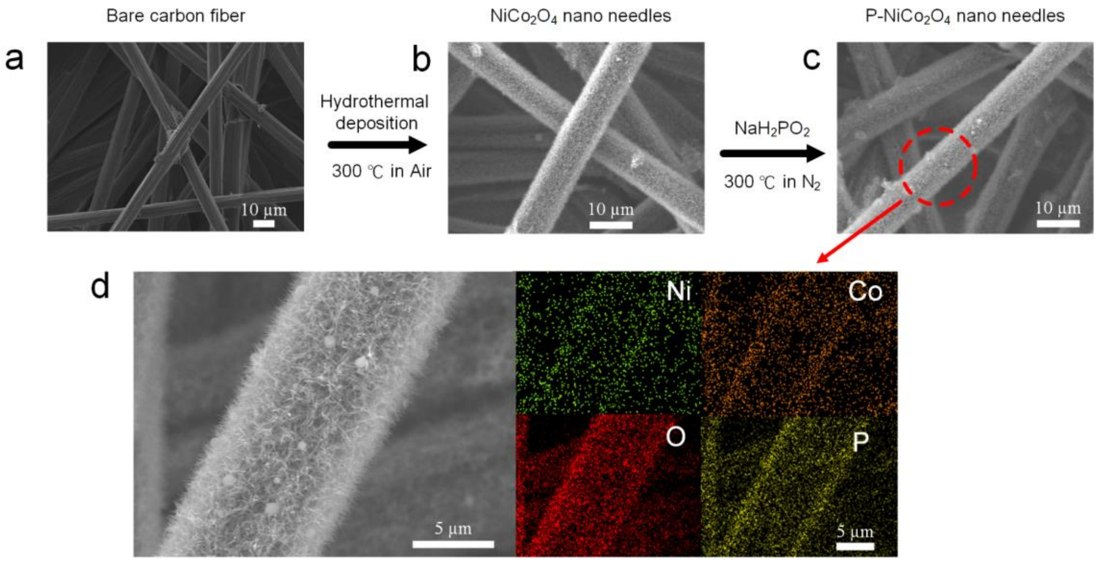

Supplementary Figure 5. Scanning electron microscopy images showing the various steps of the P-$NiCo_2O_4$ electrode active material and its nanometric needle-like structure.

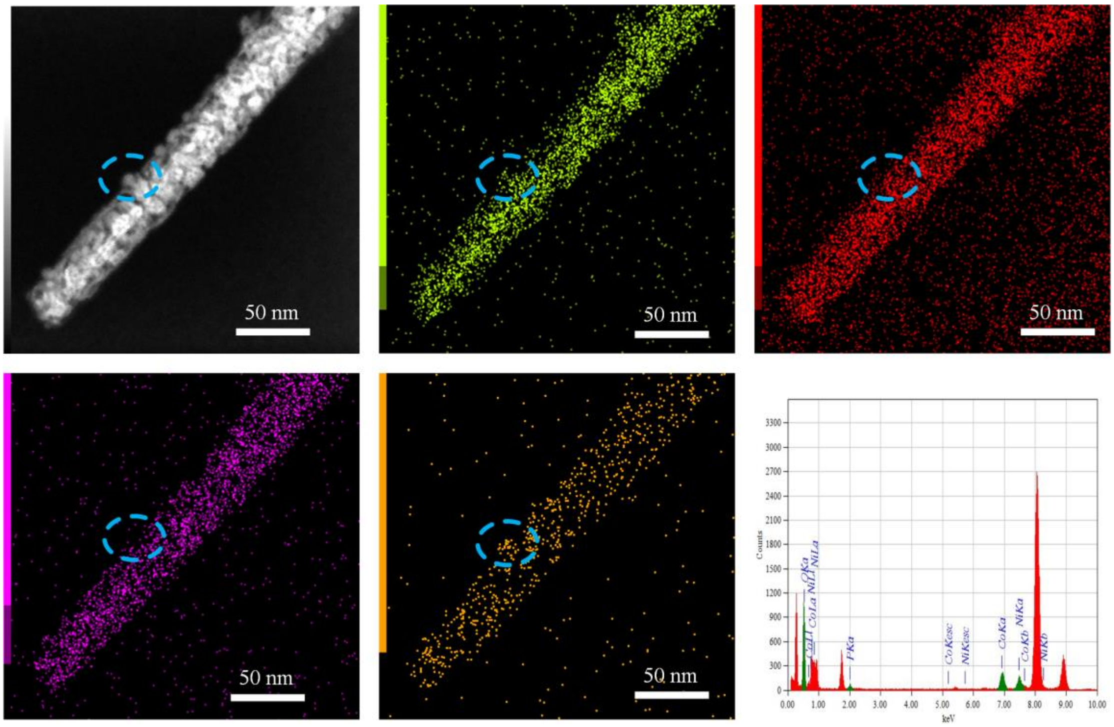

Supplementary Figure 6. EDS element distribution of Co, Ni, O, P on the P-$NiCo_2O_4$ needle-like material. Blue circle highlights a Co_2P particle, only Co and P element shows aggregation.

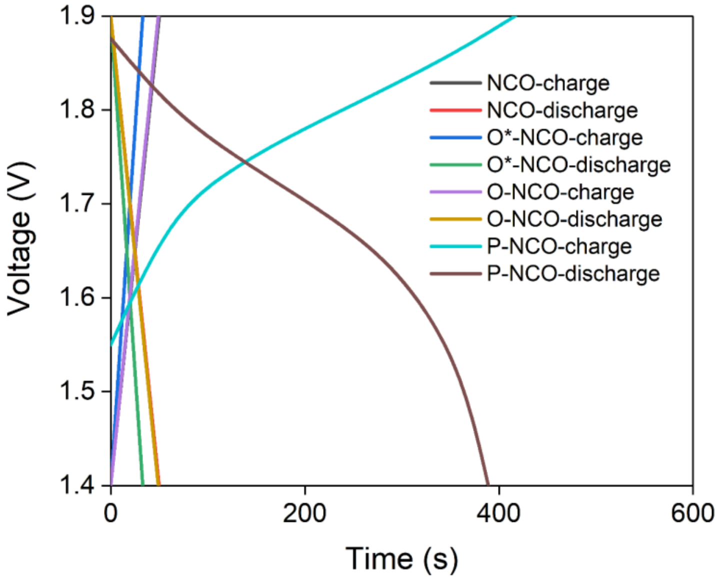

Supplementary Figure 7. Charge-discharge profiles of four $NiCo_2O_4$ (NCO) samples, O*-NCO refers to H_2/Ar annealing sample in which oxygen atoms are partially removed; O-NCO refers to O_2 plasma treated sample; P-NCO refers to phosphatized sample. Specific current is 2 A g^{-1}.

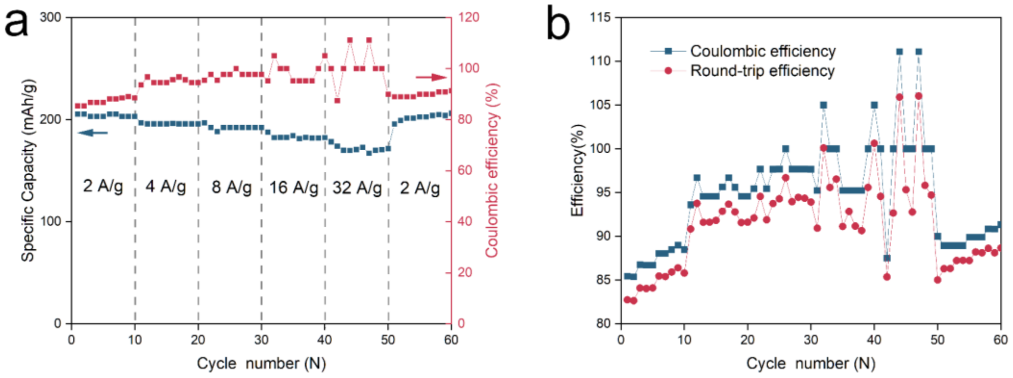

Supplementary Figure 8. (a) Specific capacity and (b) Coulombic efficiency delivered by the aqueous Zn||Co_2P-CoP-$NiCoO_2$ cell during rate capability test

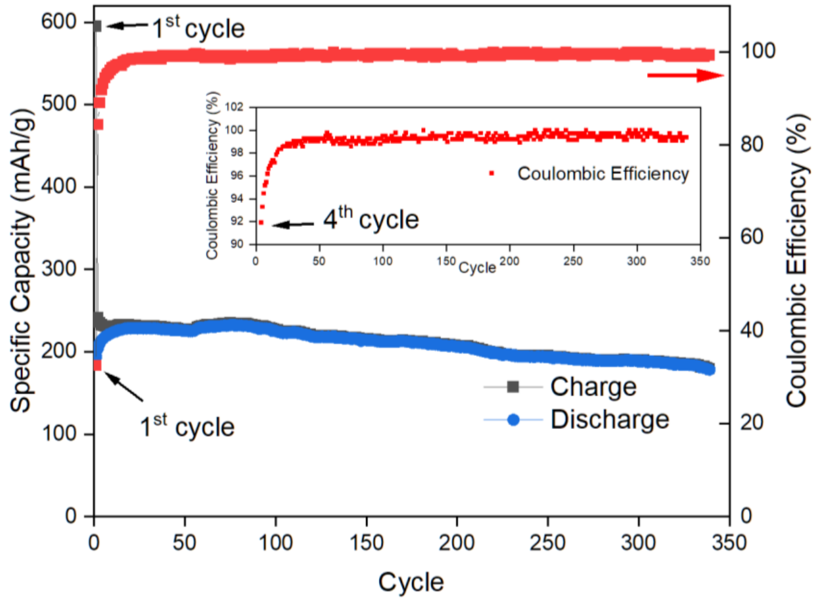

Supplementary Figure 9. Cycling performance of the aqueous Zn||Co_2P-CoP-$NiCoO_2$ cell at a specific current of 2 A/g.

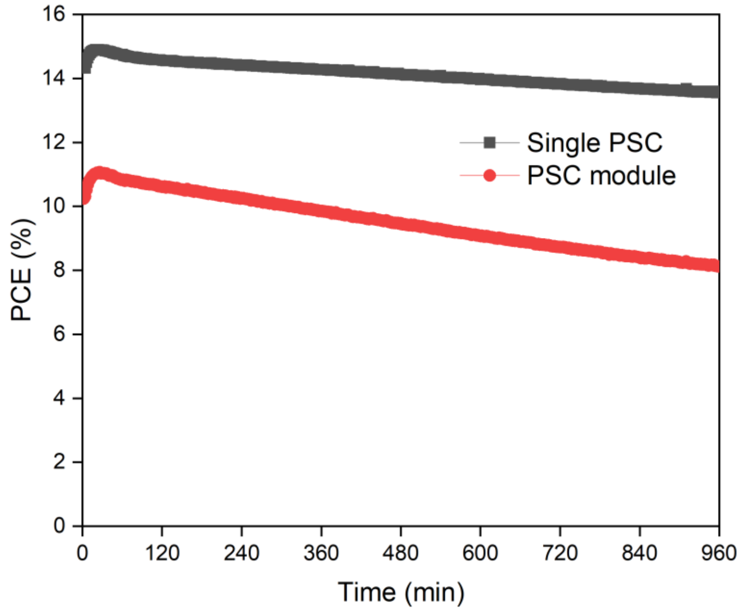

Supplementary Figure 10. PCE performance of single PSC and three PSC connected PSC module under 1000 minutes continuous AM 1.5 illumination in air without encapsulation.

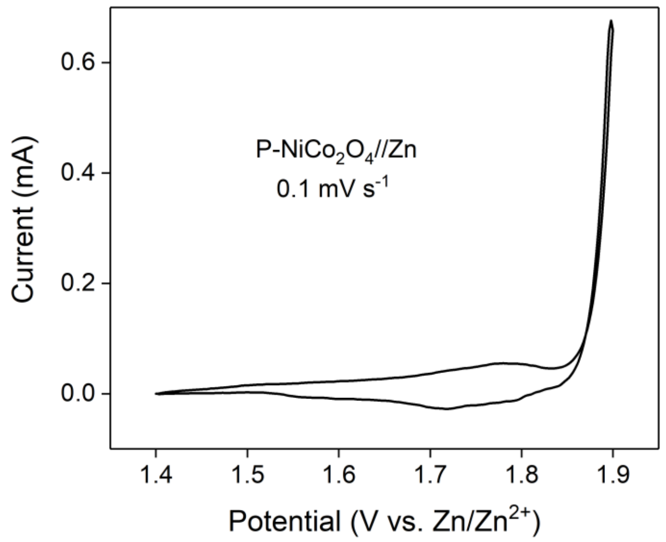

Supplementary Figure 11. Cyclic voltammetry tests of the aqueous Zn||P-$NiCo_2O_4$ cell from 1.4-1.9 V at 0.1 mV s^{-1}.

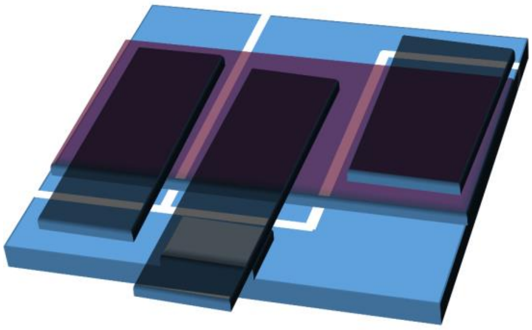

Supplementary Figure 12. Three perovskite solar cells connected in series on a single substrate.

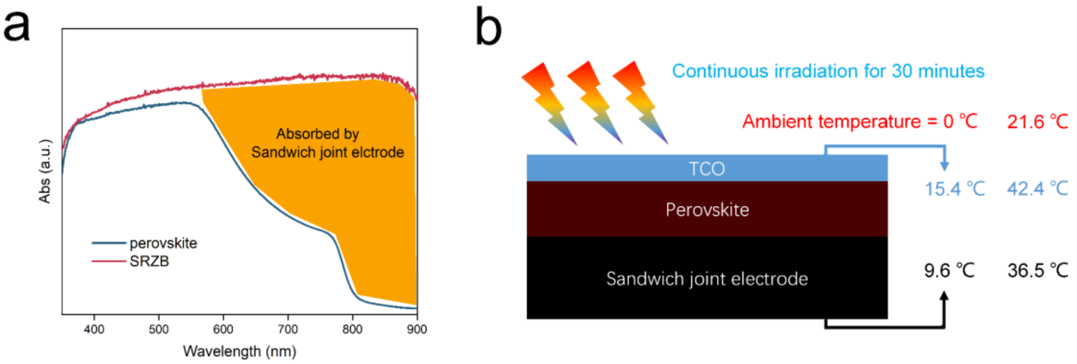

Supplementary Figure 13 (a) UV-Vis absorption spectra of SRZB, perovskite and Sandwich joint electrode. (b) Photothermal effect of light illumination.

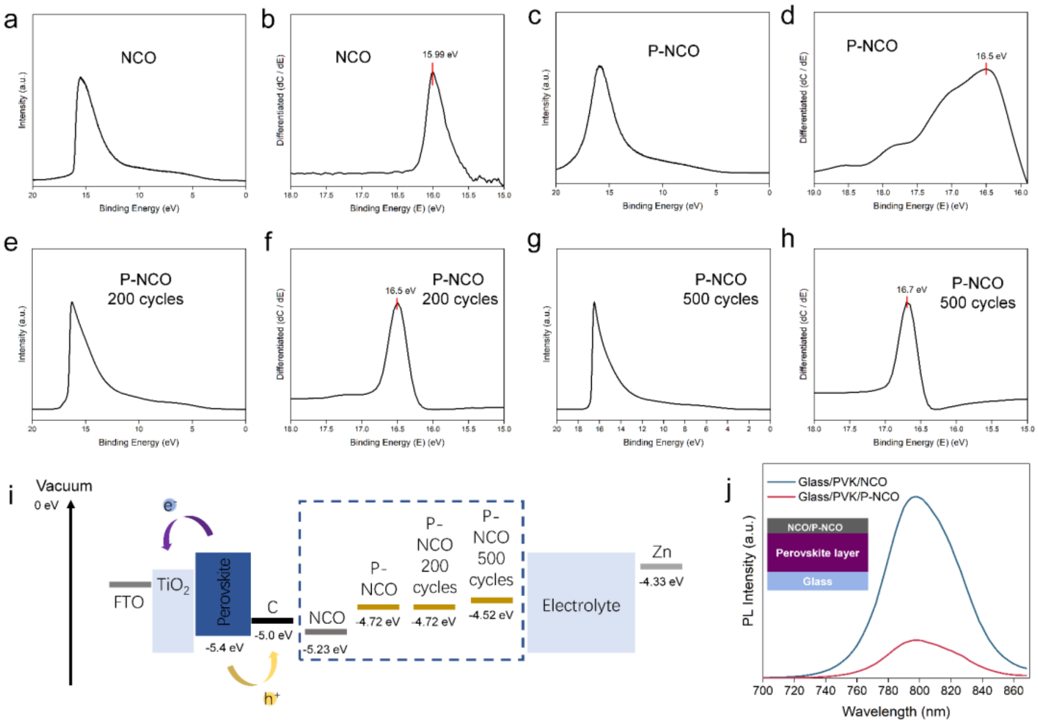

Supplementary Figure 14 (a-b) Ultraviolet Photoelectron Spectroscopy (UPS) measurements of $NiCo_2O_4$ (NCO). (c-d) UPS of P-NCO. (e-f) UPS of P-NCO after 200 cycles (8A/g). (g-h) UPS of P-NCO after 500 cycles (8A/g). (i) Band structure of solar rechargeable zinc battery (versus vacuum energy level at 0 eV), the data of perovskite, carbon and Zn is from open literatures. (j) Photoluminescence (PL) measurement of glass/perovskite/NCO and glass/perovskite/P-NCO.

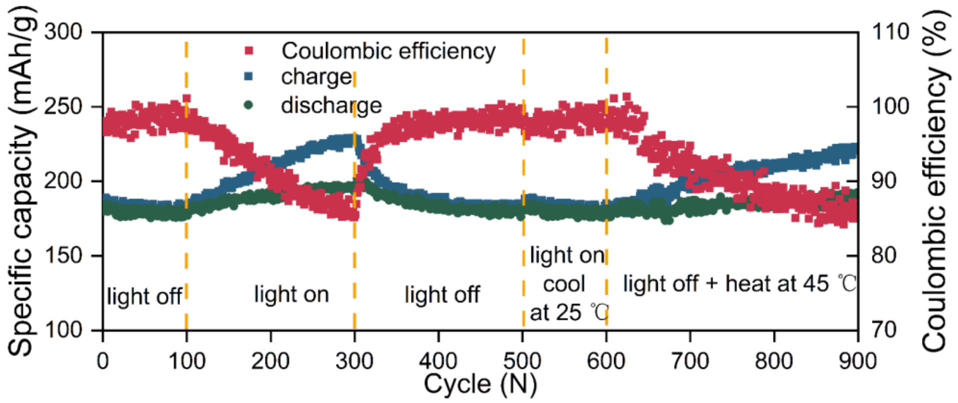

Supplementary Figure 15 Cycling performance of the aqueous Zn||Co_2P-CoP-$NiCoO_2$ cell at a specific current of 32 A/g under different light off, light on, cooling and heating conditions.

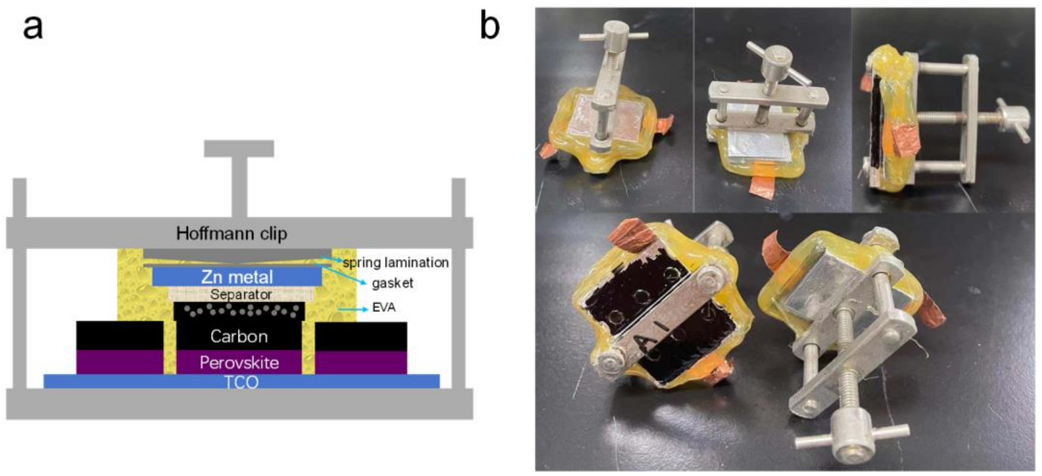

Supplementary Figure 16. (a) Graphical representation and (b) Digital images of the solar rechargeable zinc battery. All the metal surfaces which may reflect light and influence the results have been covered.

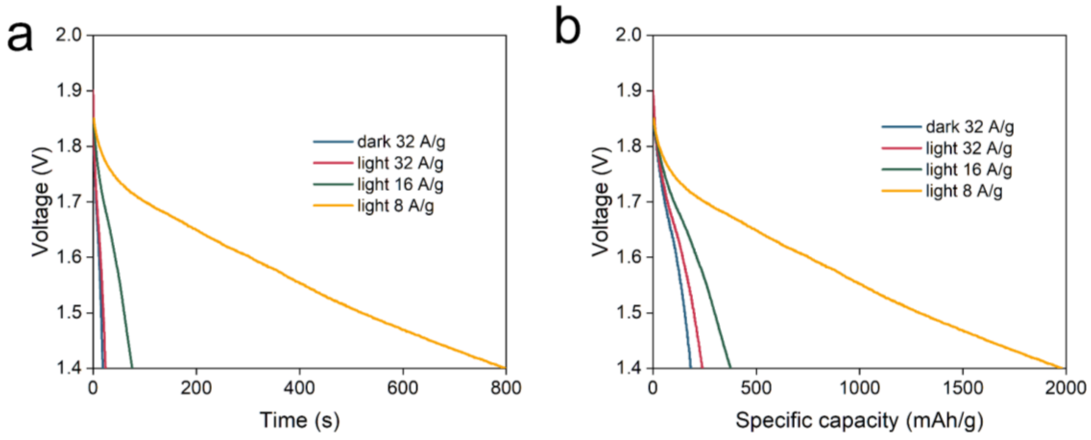

Supplementary Figure 17. Discharging capability of the photo-rechargeable battery-integrated device under various specific currents and light conditions

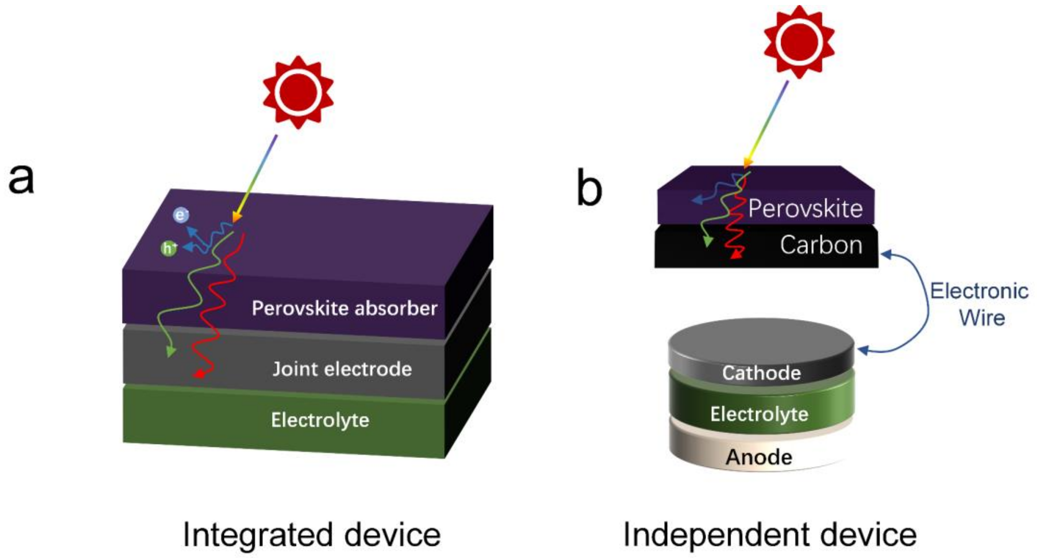

Supplementary Figure 18. Schematic diagram of integrated and independent solar rechargeable devices.

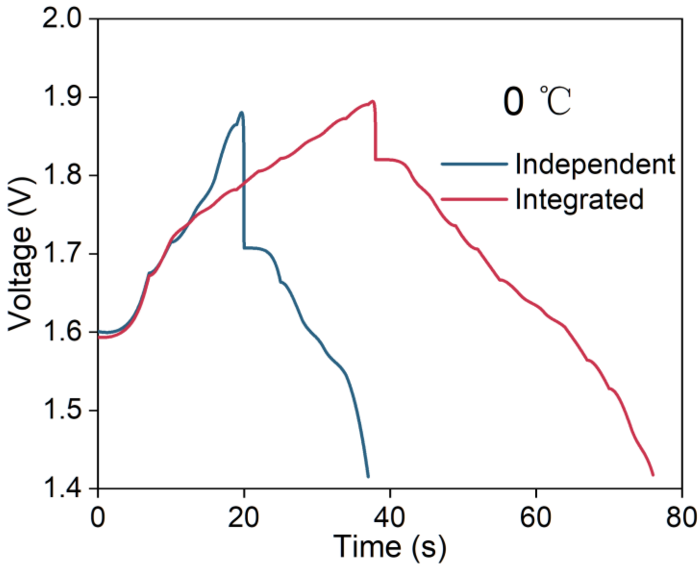

Supplementary Figure 19. Photo-assisted charge/discharge of the solar rechargeable zinc battery at a specific current of 8A/g at 0 °C. Independent structure and integrated structure see Supplementary Figure 18.

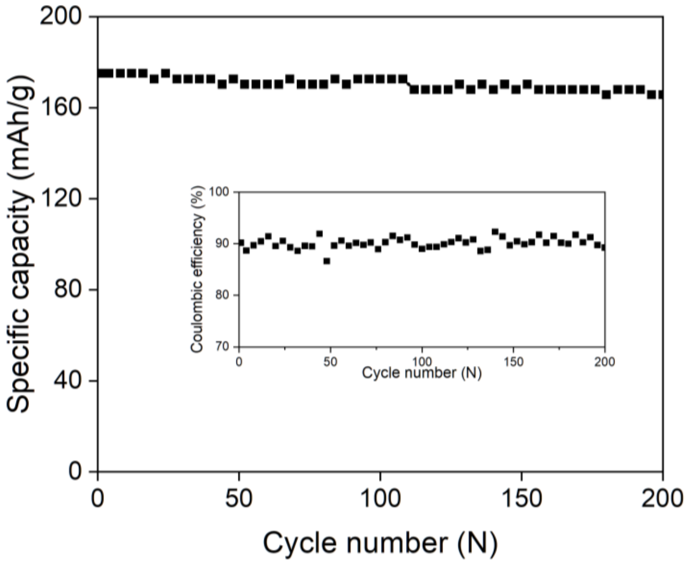

Supplementary Figure 20. Specific discharge capacity of solar rechargeable zinc battery during 200 photo-charge and galvanostatic discharge cycles (0.42 cm^2 and 8A/g). Inset is the coulombic efficiency of solar rechargeable zinc battery during photo-charge and galvanostatic discharge with photothermal induced temperature at around 36.5 °C.

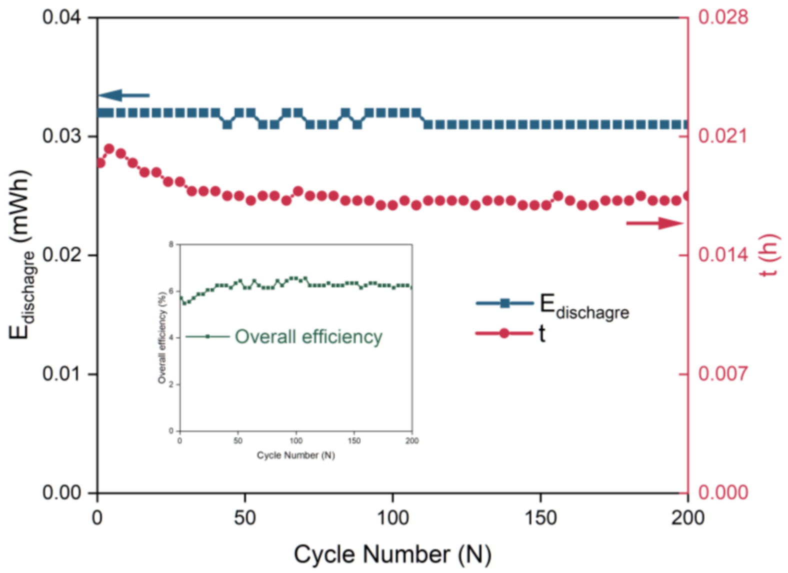

Supplementary Figure 21. $E_{discharge}$, t and η trendency of SRZB during 200 cycles.
