# Supplementary material for: Coupling aqueous zinc batteries and perovskite solar cells for simultaneous energy harvest, conversion and storage
Source: Nat Commun. 2022 Jan 10;13:64. doi: 10.1038/s41467-021-27791-7 (PMC8748727; doi:10.1038/s41467-021-27791-7)
Supplement: Supplementary file 3 — Reporting Summary [file 41467_2021_27791_MOESM3_ESM.pdf]

nature portfolio

Corresponding author(s): Gao Xueping

Last updated by author(s): Dec 2, 2021

Reporting Summary

Nature Portfolio wishes to improve the reproducibility of the work that we publish. This form provides structure for consistency and transparency in reporting. For further information on Nature Portfolio policies, see our Editorial Policies and the Editorial Policy Checklist.

Statistics

For all statistical analyses, confirm that the following items are present in the figure legend, table legend, main text, or Methods section.

n/a	Confirmed	
☒	☐	The exact sample size (*n*) for each experimental group/condition, given as a discrete number and unit of measurement
☒	☐	A statement on whether measurements were taken from distinct samples or whether the same sample was measured repeatedly
☒	☐	The statistical test(s) used AND whether they are one- or two-sided<br>*Only common tests should be described solely by name; describe more complex techniques in the Methods section.*
☒	☐	A description of all covariates tested
☒	☐	A description of any assumptions or corrections, such as tests of normality and adjustment for multiple comparisons
☒	☐	A full description of the statistical parameters including central tendency (e.g. means) or other basic estimates (e.g. regression coefficient) AND variation (e.g. standard deviation) or associated estimates of uncertainty (e.g. confidence intervals)
☒	☐	For null hypothesis testing, the test statistic (e.g. *F*, *t*, *r*) with confidence intervals, effect sizes, degrees of freedom and *P* value noted<br>*Give P values as exact values whenever suitable.*
☒	☐	For Bayesian analysis, information on the choice of priors and Markov chain Monte Carlo settings
☒	☐	For hierarchical and complex designs, identification of the appropriate level for tests and full reporting of outcomes
☒	☐	Estimates of effect sizes (e.g. Cohen's *d*, Pearson's *r*), indicating how they were calculated

Our web collection on statistics for biologists contains articles on many of the points above.

Software and code

Policy information about availability of computer code

Data collection	no software was used
Data analysis	Origin, EXCEL, Avantage, Jade, and GMS3 was used for analysis and plot drawing

For manuscripts utilizing custom algorithms or software that are central to the research but not yet described in published literature, software must be made available to editors and reviewers. We strongly encourage code deposition in a community repository (e.g. GitHub). See the Nature Portfolio guidelines for submitting code & software for further information.

Data

Policy information about availability of data

All manuscripts must include a data availability statement. This statement should provide the following information, where applicable:

- Accession codes, unique identifiers, or web links for publicly available datasets
- A description of any restrictions on data availability
- For clinical datasets or third party data, please ensure that the statement adheres to our policy

The data that support the findings of this study are available from the authors on reasonable request.

Field-specific reporting

Ecological, evolutionary & environmental sciences study design

All studies must disclose on these points even when the disclosure is negative.

Study description	Study the new type solar rechargeable zinc battery driven by perovskite solar cells, the main topic is about green energy conversion and storage.
Research sample	Integrated solar rechargeable zinc battery, as well as the individual zinc battery and perovskite solar cells.
Sampling strategy	We took the preliminary tests of individual zinc battery and perovskite solar cells for more than 100 samples, after optimization we fabricated the integrated solar rechargeable zinc battery for tens of samples. Since our work is mainly about a new conception, we believe such amount of data is sufficient.
Data collection	Data was collected from Land tester for electrochemical tests, Keithley 2400 source meter for JV tests. All the photo charging and discharge experiments were controlled manually. Detailed measurement condition is provided in the manuscript.
Timing and spatial scale	From 2020.5.17 to 2021.10.31, in Tianjin, China.
Data exclusions	No data was excluded.
Reproducibility	During the past 18 months, we have repeated the experiments for many times, we believe other groups could also repeat it following our experimental conditions.
Randomization	Solar cells and joint electrode samples were chosen randomly for testing and fabricating the integrated solar rechargeable zinc battery.
Blinding	not relevant

Did the study involve field work? ☐ Yes ☒ No

Reporting for specific materials, systems and methods

We require information from authors about some types of materials, experimental systems and methods used in many studies. Here, indicate whether each material, system or method listed is relevant to your study. If you are not sure if a list item applies to your research, read the appropriate section before selecting a response.

Materials & experimental systems

n/a	Involved in the study
☒	☐ Antibodies
☒	☐ Eukaryotic cell lines
☒	☐ Palaeontology and archaeology
☒	☐ Animals and other organisms
☒	☐ Human research participants
☒	☐ Clinical data
☒	☐ Dual use research of concern

Methods

n/a	Involved in the study
☒	☐ ChIP-seq
☒	☐ Flow cytometry
☒	☐ MRI-based neuroimaging
