# Supplementary material for: Coupling aqueous zinc batteries and perovskite solar cells for simultaneous energy harvest, conversion and storage
Source: Nat Commun. 2022 Jan 10;13:64. doi: 10.1038/s41467-021-27791-7 (PMC8748727; doi:10.1038/s41467-021-27791-7)
Supplement: Supplementary file 4 — Solar Cells Reporting Summary [file 41467_2021_27791_MOESM4_ESM.pdf]

nature research

Corresponding author(s): Gao Xueping

Solar Cells Reporting Summary

Nature Research wishes to improve the reproducibility of the work that we publish. This form is intended for publication with all accepted papers reporting the characterization of photovoltaic devices and provides structure for consistency and transparency in reporting. Some list items might not apply to an individual manuscript, but all fields must be completed for clarity.

For further information on Nature Research policies, including our data availability policy, see Authors & Referees.

ü Experimental design

Please check: are the following details reported in the manuscript?

1. Dimensions

Item	Yes/No	Details
Area of the tested solar cells	[x] Yes [ ] No	The area is 0.1 cm2 for solar cells and 0.42 cm2 for SRZB, as illustrated in the Section of "Methods" in the Manuscript.
Method used to determine the device area	[x] Yes [ ] No	The device area is defined by using mask, and this information is illustrated in Section of "Methods" in the manuscript

2. Current-voltage characterization

Item	Yes/No	Details
Current density-voltage (J-V) plots in both forward and backward direction	[ ] Yes [x] No	Backward scan is applied for preliminary characterization, and overall efficiency is directly calculated from solar energy and discharge electrical energy.
Voltage scan conditions<br>*For instance: scan direction, speed, dwell times*	[x] Yes [ ] No	The scan direction is 1.2 V to -0.1 V, with a scan step of 5 mV and dwell time is 1 ms, described in the section of "Methods" in the Manuscript.
Test environment<br>*For instance: characterization temperature, in air or in glove box*	[x] Yes [ ] No	Devices were tested in Air condition, and this information is illustrated in "Method" section in the revised manuscript.
Protocol for preconditioning of the device before its characterization	[ ] Yes [x] No	No preconditioning protocol
Stability of the J-V characteristic<br>*Verified with time evolution of the maximum power point or with the photocurrent at maximum power point; see ref. 7 for details.*	[x] Yes [ ] No	Shown in supplementary figure 10.

3. Hysteresis or any other unusual behaviour

Item	Yes/No	Details
Description of the unusual behaviour observed during the characterization	[ ] Yes [x] No	No unusual behaviour.
Related experimental data	[ ] Yes [x] No	No unusual behaviour.

4. Efficiency

Item	Yes/No	Details
External quantum efficiency (EQE) or incident photons to current efficiency (IPCE)	[ ] Yes [x] No	The focus of this work is not about solar cells, such information is not requisite.
A comparison between the integrated response under the standard reference spectrum and the response measure under the simulator	[ ] Yes [x] No	The focus of this work is not about solar cells, such information is not requisite.
For tandem solar cells, the bias illumination and bias voltage used for each subcell	[ ] Yes [x] No	No tandem solar cells are fabricated in our work.

5. Calibration

Item	Yes/No	Details
Light source and reference cell or sensor used for the characterization	[x] Yes [ ] No	The light source is a Class 2A solar simulator (Oriel Sol2A, Newport). The light intensity is calibrated with a 20 mm×20 mm monocrystalline silicon reference cell (purchased from Newport). The details can be found in the Section of "Methods" in the manuscript.

Confirmation that the reference cell was calibrated and certified	[x] Yes<br>[ ] No	The standard monocrystalline silicon reference cell was calibrated and certified. The details can be found in the Section of "Methods" in the manuscript.
Calculation of spectral mismatch between the reference cell and the devices under test	[ ] Yes<br>[x] No	We did not calculate the mismatch between the reference cell and the devices under test, for this value is very small with our solar simulator and calibration reference cell.

6. Mask/aperture

Size of the mask/aperture used during testing	[x] Yes<br>[ ] No	A mask with an area of 0.1 cm2 and 0.42 cm2 was used during testing, as illustrated in "Method" section.
Variation of the measured short-circuit current density with the mask/aperture area	[ ] Yes<br>[x] No	The focus of this work is not about solar cells, such information is not requisite.

7. Performance certification

Identity of the independent certification laboratory that confirmed the photovoltaic performance	[ ] Yes<br>[x] No	The focus of this work is not about solar cells, such information is not requisite.
A copy of any certificate(s)<br>*Provide in Supplementary Information*	[ ] Yes<br>[x] No	The focus of this work is not about solar cells, such information is not requisite.

8. Statistics

Number of solar cells tested	[ ] Yes<br>[x] No	We have tested a lot of cells during the experiment, but the focus of this work is not about solar cells, so we haven't analyzed those data.
Statistical analysis of the device performance	[ ] Yes<br>[x] No	We have tested a lot of cells during the experiment, but the focus of this work is not about solar cells, so we haven't analyzed those data.

9. Long-term stability analysis

Type of analysis, bias conditions and environmental conditions<br>*For instance: illumination type, temperature, atmosphere humidity, encapsulation method, preconditioning temperature*	[x] Yes<br>[ ] No	Irradiance stability under AM 1.5 G illumination in air was tested for unencapsulated devices. The results are provided in Fig. 2c and Supplementary Figure 10.
